# Primary aorto‐enteric fistula diagnosed by double‐balloon endoscopy

**DOI:** 10.1002/deo2.70118

**Published:** 2025-04-16

**Authors:** Momoko Yamamoto, Kei Nomura, Tomoyoshi Shibuya, Masashi Omori, Rina Odakura, Kentaro Ito, Hirofumi Fukushima, Osamu Nomura, Dai Ishikawa, Akihito Nagahara

**Affiliations:** ^1^ Department of Gastroenterology Juntendo University School of Medicine Japan; ^2^ Department of Pathophysiological Research and Therapeutics for Gastrointestinal Disease Juntendo University School of Medicine Japan

**Keywords:** abdominal aortic aneurysm, antegrade double‐balloon endoscopy, herald bleeding, jejunum, primary aorto‐enteric fistula

## Abstract

A primary aorto‐enteric fistula (PAEF), rarer than a secondary aorto‐enteric fistula, is a direct rupture of the bowel by an abdominal aortic aneurysm (AAA). More than 54% of cases were in the duodenum, while jejunum and ileum were affected in only 15% of cases.

An 82‐year‐old woman with hematemesis and hematochezia was admitted to our hospital emergently. Upper and lower endoscopies did not reveal the source of bleeding. We performed an urgent antegrade double‐balloon endoscopy, revealing a submucosal tumor‐like protuberance with an ulcer in the jejunum. These findings raised suspicion by PAEF. Subsequent computed tomography showed free air near the AAA, confirming PAEF as the hemorrhage source. An abdominal aortic stent graft was implanted, followed by laparotomy. An adhesion between the AAA and small intestinal wall was found. Postoperative recovery was uneventful, with no recurrence observed. This case underscores the importance of considering PAEF as a potential diagnosis in patients with gastrointestinal bleeding and a history of AAA. Endoscopists should be aware of submucosal tumor such as in this case to avoid misdiagnosing PAEF, as diagnosis and intervention are crucial for favorable outcomes.

## INTRODUCTION

Aorto‐enteric fistula (AEF) where the aorta and gastrointestinal tract perforate and cause gastrointestinal bleeding, is an extremely serious condition that can lead to fatal major bleeding.^1^ AEF can be classified as primary AEF (PAEF) and secondary AEF (SAEF). The majority of PAEF causes are the presence of an abdominal aortic aneurysm (AAA), and other causes include bacterial infection, radiotherapy, cystic media necrosis, and temporal arteritis are possible. On the other hand, SAEF causes include crush necrosis due to direct contact between the artificial blood vessel and the intestinal tract after abdominal revascularization, and dehiscence of the anastomosis due to the spread of infection.^2^, ^3^ The most common organ for PAEF perforation is the third portion of the duodenum in 54%, followed by the esophagus in 28%, the small intestine in 15%, and the stomach in 2%.^4^

This case represents the first reported instance of PAEF in jejunum diagnosed using antegrade double‐balloon endoscopy (DBE), and we present it here with a comprehensive discussion.

## CASE REPORT

**FIGURE 1:**
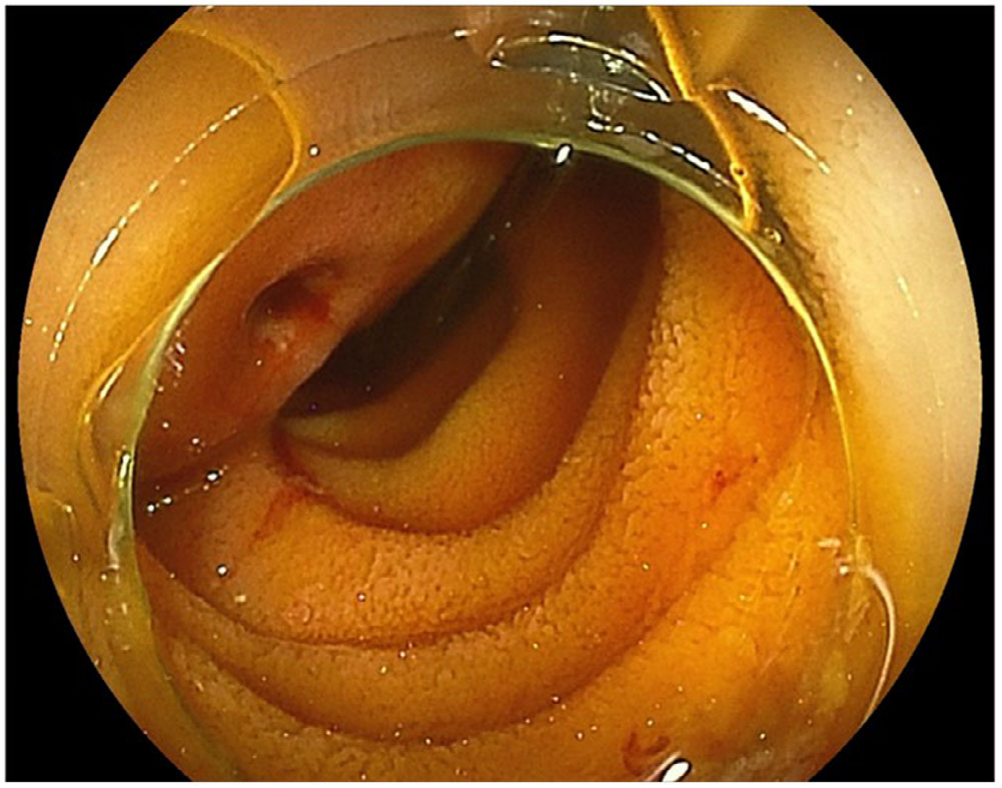
Anterograde double‐balloon endoscopy revealed a submucosal tumor‐like elevation with an ulcer 50 cm above the ligament of Treitz, consistent with the site of an abdominal aortic aneurysm.

**FIGURE 2:**
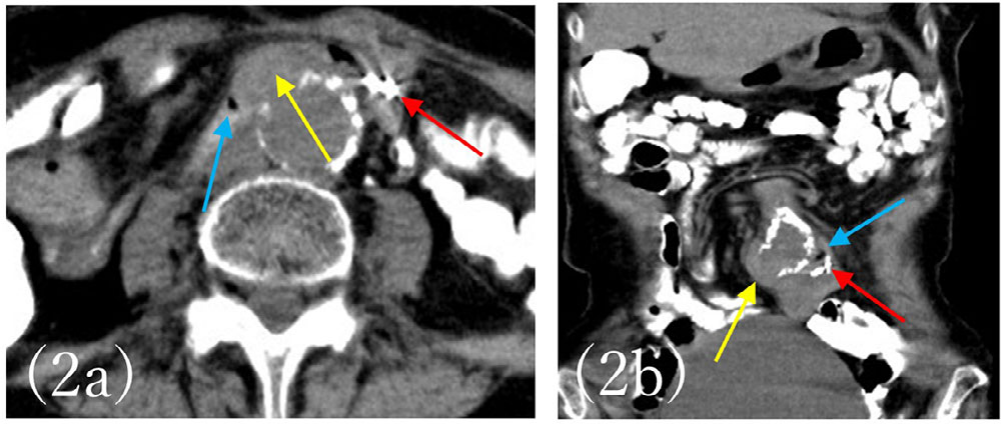
Axial plane (a) and coronal plane (b) of the computed tomography (scan showed the abdominal aortic aneurysm. A 35 mm aneurysm was found in the lower abdominal aorta at the bifurcation of the renal arteries. Blue arrows indicate free air, yellow arrows indicate saccular protrusion from the aorta toward the small intestine, and red arrows indicate marking clips.

**FIGURE 3:**
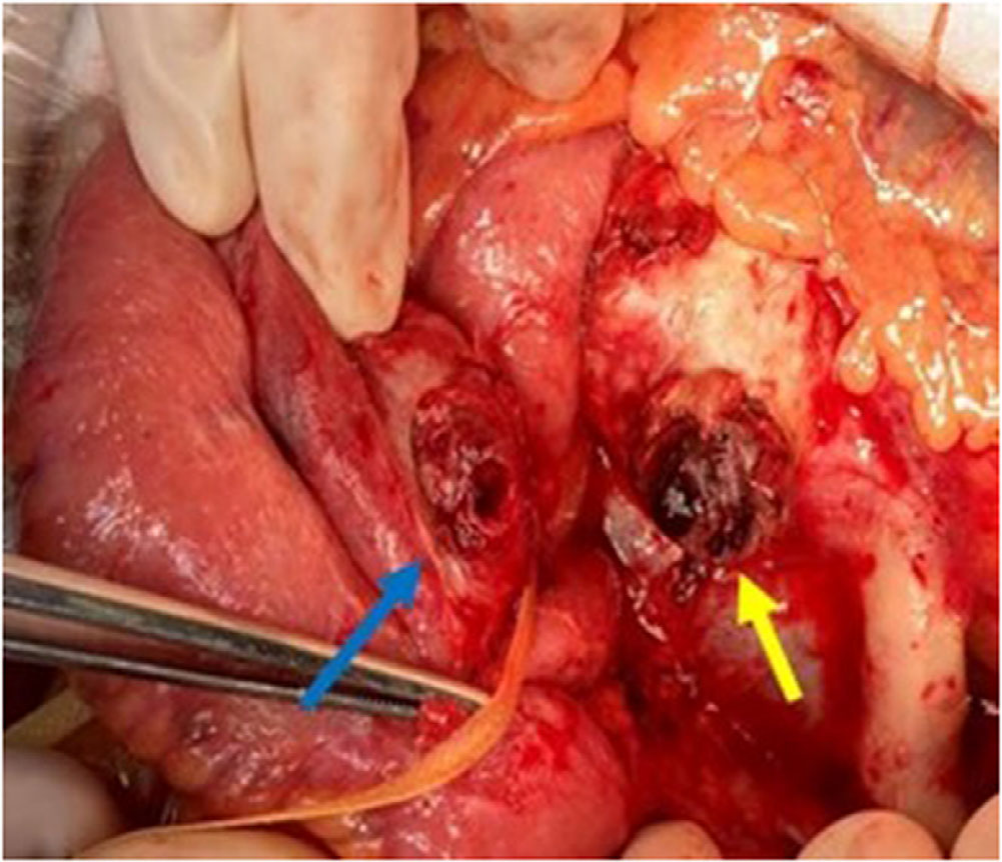
External view of the affected jejunum loop and the aneurysmatic aortic defect. The blue arrow indicates the area of the defect in the jejunum. The yellow arrow indicates the dissected fistula site of the abdominal aortic aneurysm.

An 82‐year‐old woman had been hospitalized at another hospital for untreated AAA, myocardial infarction, and ovarian cancer which had been treated with surgery, chemotherapy, and radiation therapy. She vomited a small amount of dark red blood, but it was difficult to treat her at the hospital, therefore she was consulted to our hospital. At the time of admission, her vital signs were stable: blood pressure 120/65 mmHg, pulse rate 75/min, respiration rate 16/min. Blood sampling revealed mild normocytic anemia with hemoglobin (Hb) of 10.5 g/dL and mean corpuscular volume of 91.6 fL, and no discrepancy in blood urea nitrogen (BUN)/Creatin (Cre) with BUN of 14 mg/dL and Cre of 0.70 mg/dL  (Table 1). Abdominal contrast‐enhanced computed tomography (CT) was performed, but the size of 35 mm AAA was found, and no contrast medium leaked into the gastrointestinal tract. Since she was taking aspirin/vonoprazan fumarate after a myocardial infarction, it was determined that the risk of bleeding was high, and she was admitted to the hospital. Esophagogastroduodenoscopy (EGD) was performed on the same day of admission, and no obvious source of bleeding could be identified. However, the next day, she lost consciousness and had a temporary drop in blood pressure, and a colonoscopy was performed. Accumulation of dark red stool from the small intestine to the large intestine was found and we suspected lower small intestine bleeding. After that, retrograde DBE was performed and inserted into the small intestine 2.5 meters from the Bauhin valve, but the source of bleeding could not be identified. Thereafter, there was no rebleeding, therefore eating and aspirin/vonoprazan were resumed. It was confirmed that there was no progression of anemia. She had hematemesis of fresh blood, and Hb had fallen to 7.0 g/dL. Upper gastrointestinal bleeding was suspected again and EGD was performed. Although no gross lesions were observed from the duodenum to the esophagus, there was a large amount of black residue up to the duodenum, and antegrade DBE was performed in consideration of the possibility of upper small intestine bleeding. Antegrade DBE showed SMT‐like protuberance with an ulcer (Figure 1). A marking clip was placed near the pulsatile submucosal tumor (SMT)‐like elevation. The CT scan taken after DBE showed a cystic protrusion and free air from the AAA toward the small intestine, and the position of the clip and AAA were consistent with each other (Figure 2), leading to the diagnosis that PAEF was the cause of the hemorrhage. Cardiovascular surgery and colorectal surgery were planned for AEF and an impending rupture of AAA was suspected. First, an abdominal aorta stent graft was inserted and then a laparotomy was performed at the colorectal surgery department. Observation of the small intestine revealed AAA and adhesions between the small intestine wall 50 cm from the Treitz ligament (Figure 3). After removing the adhesions between the aortic aneurysm and the small intestine wall, removing the thrombus from the aortic aneurysm, and closing the fistula, functional end‐to‐end anastomosis was performed for the small intestine fistula, and the surgery was completed. The postoperative course was uneventful, and she was discharged from the hospital, there has been no recurrence even now after the operation.

**TABLE 1:** Laboratory findings on admission.

WBC	5600	10^9^/L	CHE	69	U/L
RBC	344	10^12^/L	TP	6.0	g/dL
Hb	10.5	g/dL	Alb	3.6	g/dL
Hct	31.5	%	BUN	14	mg/dL
MCV	92.6	fL	Cre	0.70	mg/dL
RET	2.3	%	Na	143	mmol/L
Plt	121	10^9^/L	K	4.3	mmol/L
PT	81	%	Cl	108	mmol/L
D‐dimer	3.8	µg/dL	Fe	215	µg/dL
ALP	84	U/L	Ferritin	88	ng/dL
AST	14	U/L	TIBC	231	µg/dL
ALT	9	U/L	CRP	0.07	mg/dL
LD	160	U/L	CEA	1.7	ng/dL
GGT	13	U/L	CA125	7	U/mL
CK	55	U/L	CA19‐9	9	U/mL

Abbreviations: Alb, Albumin; ALP, Alkaline Phosphatase; ALT, Alanine Aminotransferase; AST, Aspartate Aminotransferase; BUN, Blood Urea Nitrogen; CA125, Cancer Antigen 125; CA19‐9, Cancer Antigen 19‐9; CEA, Carcinoembryonic Antigen; CHE: Cholinesterase; CK, Creatine Kinase; Cl, Chloride; Cre, Creatinine; CRP, C‐Reactive Protein; Fe, Iron; GGT, Gamma‐Glutamyl Transpeptidase; Hb, Hemoglobin; Hct, Hematocrit; K, Potassium; LD, Lactate Dehydrogenase; MCV, Mean Corpuscular Volume; Na, Sodium; Plt, Platelet count; PT, Prothrombin Time; RBC, Red Blood Cell count; RET, Reticulocyte count; TIBC, Total Iron Binding Capacity; TP, Total Protein; WBC, White Blood Cell count.

## DISCUSSION

AEF is a rare disease in which a fistula is formed between the aorta and the intestine. AEF is a fatal disease with a high mortality rate of 40%–70%.^5^ It is classified into PAEF which the aorta communicates directly with the intestine, and SAEF which occurs after graft grafting.^2^, ^3^ This case was PAEF because she had untreated AAA. Because PAEF occurs between the third portion of the duodenum, jejunum, and ileum near the aorta, it is thought to be caused by pulsatile compression of the intestinal wall by the aneurysm wall. Since she was followed up at another hospital, the details are unclear, she had untreated AAA for a long time and the fistula was most likely caused by pulsatile pressure. However, AAA is considered an indication for treatment when its size exceeds 45 mm in females or 50 mm in males, or when symptomatic enlargement occurs at a rate of 5 mm or more over 6 months. In this case, although she had a long‐standing AAA, its size was 35 mm, falling outside the criteria for treatment. This led to its exclusion as a potential source of bleeding, contributing to the delay in diagnosis. In AEF, gastrointestinal bleeding is commonly diagnosed, and EGD is often performed; however, it is frequently found in the 3rd portion of the duodenum, making its detection rate relatively low. Previous reports in Japan have indicated that the main endoscopic findings include ulcerative lesions, whitish nodular elevations, erosion/edema, and bleeding. The fistula is temporarily closed due to a blood clot within a large aneurysm.^6^ No active bleeding is detected during the “herald bleeding” period. When the fistula is occluded, hemostasis is achieved, and hemodynamics are relatively preserved.^7^ Therefore, diagnosis is often difficult. In this case, after the initial bleeding, intermittent bleeding occurred repeatedly and she briefly went into shock, but her hemodynamics stabilized due to thrombus formation, and the final diagnosis was made by antegrade DBE. A search of PubMed revealed no case reports of PAEF diagnosed using DBE, making this the first documented case. Diagnostic methods commonly include EGD, abdominal contrast‐enhanced CT, and angiography. In recent years, preoperative diagnosis has become possible through contrast‐enhanced CT, and its usefulness has been recognized, with some reports recommending it as the initial diagnostic approach.^8^, ^9^ However in situations where the source of bleeding is unknown, it is difficult to make the decision to proceed with open surgery as diagnostic treatment. In addition to EGD and CS, considering the possibility of small bowel bleeding, the plan was to perform a capsule endoscopy on an outpatient basis once her condition stabilized. However, PAEF cases, including the present one, often involve patients who are in a state of altered consciousness or shock, and identifying the source of bleeding as quickly as possible is the highest priority. Therefore, capsule endoscopy, which requires time, may not be suitable in such cases. In the case of bleeding of unknown cause in a hemodynamically stable patient with AAA, as in this case, a detailed examination is performed using antegrade DBE is useful to diagnose AEF in addition to EGD.

In conclusion, AEF, where the aorta and gastrointestinal tract perforate causing bleeding, is a rare however potentially fatal condition. A search of PubMed revealed no case reports of PAEF diagnosed using DBE, making this the first documented case. In cases where the source of bleeding cannot be diagnosed with CT or if hemodynamically stable bleeding is associated with an abdominal aneurysm, Antegrade DBE may be useful in AEF. In this case, An SMT‐like finding was seen in the jejunum on DBE with a depression and white lichen at the apex. If the findings had been confirmed without the concept of PAEF, a biopsy might have been performed as an SMT. Endoscopists should be careful not to confuse AEF and SMT.

## CONFLICT OF INTEREST STATEMENT

None.
